# Disengagement Processes Within an Early Intervention Service for First-Episode Psychosis: A Longitudinal, Qualitative, Multi-Perspective Study

**DOI:** 10.3389/fpsyt.2020.00565

**Published:** 2020-06-12

**Authors:** Rachel Tindall, Magenta Simmons, Kelly Allott, Bridget Hamilton

**Affiliations:** ^1^Orygen, Parkville, VIC, Australia; ^2^Centre for Youth Mental Health, The University of Melbourne, Parkville, VIC, Australia; ^3^Department of Nursing, The University of Melbourne, Carlton, VIC, Australia

**Keywords:** first-episode psychosis, early intervention, qualitative research, service engagement, case-management

## Abstract

**Background:**

Specialized early intervention services for first-episode psychosis have been well established in many countries to meet the unique needs of this group. However, with high drop-out rates, these services would benefit from understanding the factors that influence a person's decision to engage with, or disengage from, them. No research has explored the experiences of engagement and disengagement over time, from the perspectives of the person who experienced a first-episode psychosis, their caregiver, and their clinician. This information is crucial to help services better respond to the needs of the people using them. The aim of this study was to understand what causes and maintains periods of disengagement from early intervention services for first-episode psychosis over time.

**Methods:**

Using a longitudinal, qualitative approach, young people, their caregivers, and their clinicians were followed through their first year with an early intervention service for first-episode psychosis in Melbourne, Australia. Qualitative interviews were completed between 3–9 weeks, 4–7 months, and 11–15 months after entry to the service (or at discharge if earlier). Trajectory analysis was used to understand the data.

**Results:**

Qualitative interviews were conducted with 24 participants (55 interviews). Young people were aged 15–24 years, came from a variety of cultural backgrounds and had various psychotic diagnoses. Three major processes were identified that, over time, led to periods of service disengagement: a mismatch between service model and individual presentation (service mismatch), a lack of shared purpose (aimless engagement), and responses to individual circumstances (reactive disengagement).

**Conclusion:**

Triangulating experiences of engagement across young people, caregivers, and clinicians allows for a comprehensive understanding of what precipitates service disengagement. This study demonstrates how early intervention services for first-episode psychosis are meeting the needs of young people and caregivers, and what areas warrant improvement. The needs of service users and patterns of disengagement vary. In turn, services must be flexible and responsive to individual circumstances. The results of this study recommend that local and international policies move away from diagnostically driven models of care, to better provide an inclusive treatment service for people with transdiagnostic mental health presentations.

## Introduction

Recent findings from an independent review of mental health services in Australia have identified that overall, mental health services are not fit for purpose (1). They are not consistently meeting the needs of the people who they were developed to serve, a finding which is replicated internationally (2). Reasons for this are complex and varied. Mental health services are a component of a health system that is overall designed to treat the characteristics of physical illnesses. There are notable distinguishing factors that should be taken into account specifically for mental health service provision, including the earlier age of first episode of illness and the need for holistic, connected, and comprehensive treatment beyond that informed by the medical model. While these factors have been identified, researched and subsequent improvements made, changes in service provision have not been consistent, timely, or appropriately financed (1).

Early intervention services (EISs) for first-episode psychosis (FEP) were developed in the 1990s in response to the need for improved service provision (3). The term psychosis refers to a group of symptoms that impact a person's understanding and experience of reality (4). A FEP has substantial impacts on well-being, and individuals with psychosis benefit greatly from targeted interventions. Research has demonstrated that EISs better meet people's needs and ultimately facilitate better outcomes for people presenting with FEP than general mental health services (5).

Internationally, EISs for FEP operate with varying levels of treatment intensity and duration (6). Taking into account that 75 percent of mental health disorders begin before the age of 25 (1), EISs provide developmentally appropriate bio-psycho-social interventions that may include medication, case-management, therapy, group programs, caregiver support, psychosocial support, and vocational support (7). In Australia, specialist multidisciplinary teams (nurses, psychologists, psychiatrists, other allied health professionals) provide treatment over the first 2–5 years following the FEP. However, engagement in interventions is often poor, with young people pre-emptively exiting EISs at rates between 6 and 60 percent (8).

Mental health service “engagement” and “disengagement” are complex constructs. There are currently no consistent, comprehensive definitions of engagement or disengagement (8). This means that research measuring and seeking to understand these constructs is often contradictory or not translatable to other settings. Encouragingly, there is a shift in the literature from engagement as a binary key performance indicator or target to be achieved, toward engagement as a continuous process requiring intervention. This shift is informed by important findings from the substantial qualitative research undertaken on this issue (9–11). For the purposes of this study, we define engagement as a dynamic construct consisting of sustained and active connection with mental health services while there is a mutually articulated need. Disengagement is understood as a break in meaningful therapeutic contact that is not mutually agreed upon, on either a temporary or permanent basis.

Our understanding of what precipitates disengagement remains vague. Quantitative research has demonstrated varying prognostic factors that may lead an individual to becoming more at-risk of disengagement, but these are not definitive and do not suggest strategies to improve engagement (12). Qualitative research is better placed to understand what leads to fractures in engagement, with findings summarized in recent systematic reviews and meta-syntheses (9–11). Factors such as disempowerment, change in clinician, and stigma are generally understood to contribute to a young person's desire to disengage from a service; however, these experiences require more focused attention to fully understand them. These studies identified gaps in the literature which impact our comprehensive understanding of engagement and disengagement, such as clinician and longitudinal experiences.

To address these gaps in our understanding on engagement and disengagement, we have conducted a larger qualitative longitudinal study. We initially sought to understand early experiences of engaging with EISs for FEP and found that young people and their caregivers value the personal and relational aspects of engagement, such as building a trusting relationship with a clinician (13). Young people and caregivers entered the EIS with varying levels of treatment participation and desire for engagement, and engagement significantly benefited from tailoring treatment to the young person's goals at this early stage of contact.

We then sought to understand clinicians' experiences of engagement. While there is extensive literature examining processes that fall within the construct of engagement, such as the therapeutic relationship and rapport, there is limited research specifically seeking to understand clinician experiences of engagement with, and disengagement from EISs for FEP (11). Our findings identified the importance of resource allocation, models of care, and the demographic characteristics of the young person or clinician (14). Clinicians described holding the ultimate responsibility for engagement, and they perceived disengagement as both episodic and something that could be altered.

Both of these studies report on engagement at a period in time using a cross-sectional approach to data collection. This is consistent with most previous qualitative studies which limits our ability to fully understand the nuances of engagement and disengagement over time. There is also limited understanding of disengagement as an episodic phenomenon that may ultimately be altered. The aim of this study, therefore, is to enhance our understanding of what initiates periods of disengagement in the first year of connection with an EIS for FEP and whether these experiences change over time, from the perspectives of the person who experienced a FEP, their caregiver and their key clinician.

## Materials and Methods

### Study Context

Longitudinal qualitative research using multi-perspective interviews allows a comprehensive understanding of a phenomenon over time, particularly focusing on any shifts in attitudes or preferences (15). This study was designed to provide a holistic view of the experience and process of disengagement, by recruiting young people themselves and the key people around them. Nine participants groups (groups included a young person, their caregiver if identified and their clinician/s) were enrolled into this longitudinal qualitative study. Data were collected between July 2016 and December 2019. The full dataset follows all participant groups until the discharge of the young person from the EIS. This paper will concentrate on the initial year of contact only, as this dataset is the most robust in terms of participant retention.

The EIS from which the participants were recruited provides treatment for young people aged 15–25 years of age in Melbourne, Australia. The EIS operates out of two geographically spaced clinics and admits young people presenting with FEP who live within defined catchment areas. A maximum of 2 years consecutive treatment may be provided at a clinic or in a community setting of the person's choice. Cognitive-behavioral case-management is offered by the key clinician alongside access to a psychosocial program, crisis team and inpatient care as needed. Clinicians can be allied health professionals such as clinical psychologists, social workers, occupational therapists, or mental health nurses; however, during the data collection period, the majority were clinical psychologists. A key clinician working full time (40 hours per week) works with approximately 20–25 young people who are at varying stages of recovery from their FEP. At the time of recruitment into the study, approximately 250 young people were receiving treatment in the EIS across the two clinic sites.

### Researcher Reflexivity

Prior to commencing the study and throughout data analysis and interpretation, we reflected on our preconceived perspectives and values. This was important in order to transparently acknowledge our positionality and to bracket perspectives when conducting inductive analysis. Our perspective, informed by our clinical and research experience, was that successfully maintained engagement with an EIS for FEP could benefit all involved. It could benefit the young person who would receive a comprehensive mental health service at a critical life stage, it could benefit caregivers who would receive support and guidance in caring for their young person and it could benefit clinicians who wanted to be useful to the person and their caregivers. We noted our values of commitment to the person's individual recovery processes, inclusivity and collaboration of all involved, and provision of excellent, evidence-based care. We acknowledged and then placed these perspectives and values aside where possible, in order to hear the real and true experiences of all participants who were in their own individual and unique engagement processes.

### Young Person Recruitment

**Figure 1:**
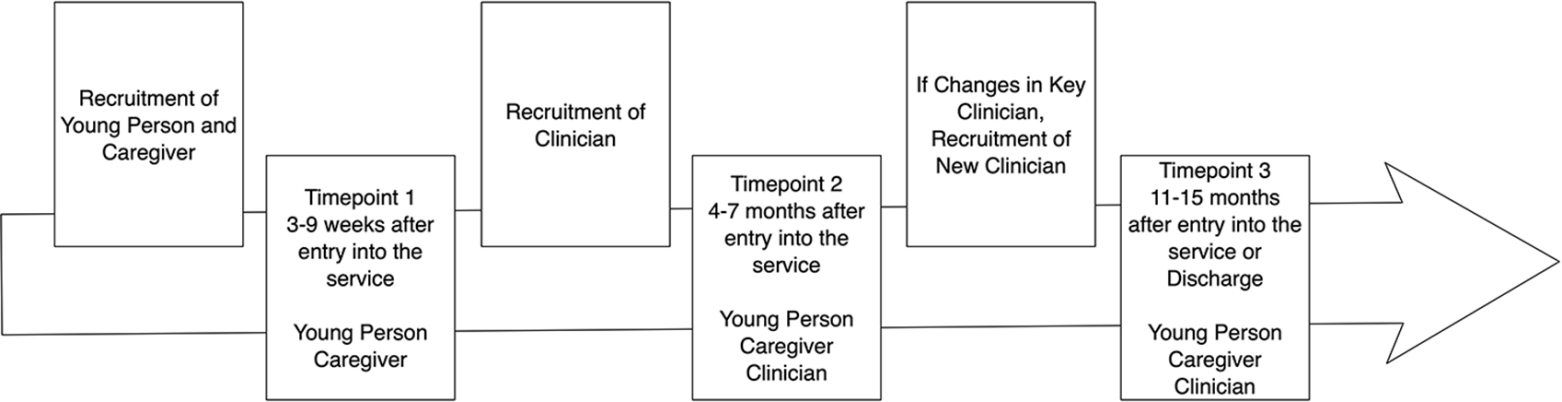
Overview of recruitment and data collection.

We attempted to recruit a “real-world” sample of young people into the study, as is summarized in our earlier paper exploring young person and caregivers' experiences of entry into an EIS (13). An overview of recruitment and data collection is provided in **Figure 1**. In summary, 45 young people who experienced a FEP and were referred into the EIS between July 2016 and March 2017 were approached to participate. Inclusion criteria were broad, with the only exclusion criterion being young people who had a clinical or personal relationship with a member of the research team, or who were unable to participate in qualitative interviews (for example, due to poor cognitive functioning). Language was not an exclusion criteria and interpreters were offered if English was a second language. An interpreter was used for one caregiver participant. Young people were introduced to the study at, or before meeting their key clinician on initial entry into the EIS. The first nine young people who consented into the study formed the nine-participant groups. We anticipated that 7–10 participant groups would be sufficient to reach data saturation, and this was confirmed through active monitoring of initial interview data.

### Participant Group Recruitment

After a young person had consented to the study, they were asked to nominate a caregiver. We anticipated that not all young people would identify as having a caregiver, as some young people live independently from their family or do not disclose their EIS attendance to their family. If a caregiver was identified, they were approached by the lead researcher and invited to participate in the study. If a caregiver was not identified or if they declined to participate in the study, the participant group continued without their data.

At the second and third time-points, the allocated key-clinician for the young person was approached and asked to participate in the study. It was expected that changes in clinician may occur over the length of the study and therefore this process was repeated for each new key clinician. If a clinician declined to participate in the study, the participant group continued without their data.

### Consent

Ethical approval was obtained from the local human research ethics committee (HREC/16/MH/131). All participants provided written informed consent. Due to the potentially fluctuating nature of psychotic symptoms and levels of distress, consent was verbally reviewed and confirmed at each interview. Consent was obtained from a parent or guardian for participants under 18 years old. If any participant withdrew from the study, consent was obtained for interviews to continue with other members of the participant group.

### Data Collection Procedures

Qualitative interviews were scheduled with participants at 8-weeks (time-point 1; young person and caregiver), 3–6 months (time-point 2; young person, caregiver and clinician), and 12–14 months (time-point 3; young person, caregiver and clinician). Contact was maintained through phone, text, or email to organize the interview, and interviews were held at a location of the participant's choice. Telephone interviews were offered as an alternative, if face-face interviews were not feasible. All clinician interviews were held at the EIS. Overall, 17 young person and 10 caregiver interviews were held at the EIS, 4 young person and 3 caregiver interviews were held at the participant's home and 3 young person interviews were conducted by phone. The lead researcher conducted all the interviews.

Demographic data, qualitative interviews, and reflective field notes were collected for all participants who participated at a time-point. All data sources are used in describing the findings of the research. Qualitative interviews were semi-structured and narrative analysis was used to understand each participant's story and the relationship/s between key players (16). Questions asked about: (1) experiences of FEP, recovery and contact with the EIS; (2) factors that pushed towards, or pulled away from engaging with the EIS, (3) relationships with other key stakeholders (young person; caregiver; clinician); and (4) roles and responsibilities in maintaining engagement. Interviews were audio recorded and transcribed verbatim. They lasted between 10 and 65 min.

All authors listened to a proportion of audio recordings to ensure that the interview process was thorough and did not introduce unnecessary bias. Written interview transcripts and a summary of the interview themes were returned to each participant for the purposes of member checking. The interview summary was also read aloud at the beginning of the next interview to allow for further member checking and to remind the participant and interviewer of themes arising from the last interview. No participant wished to change their data. Providing this summary prompted elaboration on themes and for changes in thoughts, feelings, and behaviors to be noticed and discussed during the interview.

### Data Analysis

Following member checking, all data were de-identified, and analysis commenced. Interviews were managed with a qualitative data software program (www.dedoose.com). Analysis was iterative throughout the study, which allowed themes to be explored in later interviews. The lead researcher completed all original data coding, with interviews coded thematically prior to the commencement of longitudinal trajectory analysis. Interviews were coded and analyzed both by participant group and time-point. This process is outlined in our previous papers (13, 14).

Trajectory analysis was undertaken using the method described by Grossoehme and Lipstein (15) (**Table 1**). This analysis method is most appropriate when the research aims are to understand individual experiences over time, and when the same cohort can be maintained. Using a trajectory approach allows the emphasis to be on shifts in preferences, attitudes, or explanations regarding the phenomenon, which may be missed using a cross-sectional approach. For this study, the research aims were to understand experiences of engagement with an EIS for FEP over a 12-month period, primarily focusing on flashpoints for disengagement.

**Table 1:** Application of Longitudinal Trajectory Analysis (15).

Step	Process
1	Trajectory analysis began when data was collected for all three time-points. For this study, thematic analysis of each interview had already been completed before trajectory analysis commenced. This allowed early identification of themes to inform the longitudinal matrices as described below.
2	Findings from each unit of analysis (for this study, each participant group) was mapped into its own matrix (n=9 matrices). Data was organized by broad themes along the Y-axis and time along the X-axis. The themes derived from the thematic analysis were emotions, engagement, therapeutic relationship, engagement motivators and engagement detractors.
3	A final matrix integrating the 9 trajectories was created. The focus of this matrix was how the data changed or did not change over time, across all units of analysis. In this matrix, the Y-axis was organized by themes and the X-axis was organized by participant group.
4	Data analysis was conducted from the final matrix with reference back to the first set of 9-matrices as needed. New conceptual groupings were identified as time-related concepts emerged during coding.

Researchers met frequently to discuss the emergent findings. A sample of interviews were listened to and coded by the entire research team, and all cases were discussed in depth in a 5-h workshop held to develop and refine longitudinal themes. Differences in opinion were minor, with the research team iteratively consulting the data to make any refinements to codes and themes.

## Results

### Participant Group Overview

Nine participant groups (young person, caregiver/s if applicable and key clinician/s) were followed through their first year of treatment with the EIS. There were 24 participants (9 young people; 5 caregivers; 10 clinicians) resulting in 55 qualitative interviews. There were changes in clinicians for two participants during the 12-months. One clinician was present in two case-studies and their demographic details are only reported once. Characteristics of the participants recruited to the study are given in **Table 2**. Three young people had periods of treatment under the Victorian Mental Health Act (2014) at times of acute need or high risk, but overall participants participated in the service on a voluntary legal basis. **Table 3** provides details of the interviews, including study attrition. Two young people participants experienced significant periods of complete disengagement from the EIS yet were retained in this study.

**Table 2:** Participant Characteristics.

			Total
**Young People**			**9**
	Sex (female/male)		3/6
	Age at recruitment [mean (range)]		18.4 (15–24)
	Ethnicity		
		Australian	4
		Australian/British	3
		Asian	1
		North American	1
	Diagnosis (psychotic symptoms)		
		Schizophrenia	3
		Bipolar affective disorder	2
		Depression with psychosis NOS	3
		Communication disorder	1
	Periods of treatment under the Mental Health Act at times of acute need and/or high risks		3
	Occupation		
		Student	6
		Employed	0
		Unemployed	3
	Substance use		4
	Forensic history		2
**Caregivers**			**5**
	Sex (female/male)		5/0
	Role		
		Mother	4
		Partner	1
**Clinicians**			**10**
	Sex (female/male)		7/3
	Occupation		
		Social worker	2
		Psychologist	4
		Occupational therapist	4
		Registered nurse	0
	Years since qualification		
		0–2 years	3
		3–4 years	2
		5–10 years	3
		10+ years	2

**Table 3:** Details of Interviews Conducted.

		Time-point 1	Time-point 2	Time-point 3	*Total Interviews*
		3–9 weeks	4–7 months	Discharged (n = 4): 8–12 months	
				Continuing in EIS (n = 5): 11–15 months	
Participant group 1	Young person	Interviewed at 3-weeks	Interviewed at 5-months	Discharged and interviewed at 11-months	*6 interviews*
	Caregiver	Interviewed at 3-weeks	Dropped out		
	Clinician 1a/clinician 1b		Interviewed at 5-months	Interviewed at 11-months	
Participant group 2	Young person	Interviewed at 8-weeks	Dropped out	Discharged at 8-months	*6 interviews*
	Caregiver	Interviewed at 8-weeks	Interviewed at 5-months	Interviewed at 8-months	
	Clinician		Interviewed at 5-months	Interviewed at 8-months	
Participant group 3	Young person	Interviewed at 7-weeks	Interviewed at 5-months	Interviewed at 15-months	*8 interviews*
	Caregiver	Interviewed at 7-weeks	Interviewed at 5-months	Interviewed at 15-months	
	Clinician		Interviewed at 5-months	Interviewed at 15-months	
Participant group 4	Young person	Interviewed at 8-weeks	Interviewed at 5-months	Interviewed at 14-months	*8 interviews*
	Caregiver	Interviewed at 8-weeks	Interviewed at 5-months	Interviewed at 14-months	
	Clinician 4a/clinician 4b		Interviewed at 5-months	Interviewed at 14-months	
Participant group 5	Young person	Interviewed at 8-weeks	Interviewed at 4-months	Discharged and interviewed at 8-months	*5 interviews*
	*No caregiver identified*				
	Clinician		Interviewed at 4-months	Interviewed at 8-months	
Participant group 6	Young person	Interviewed at 7-weeks	Interviewed at 4-months	Interviewed at 11-months	*5 interviews*
	*No caregiver identified*				
	Clinician		Interviewed at 4-months	Interviewed at 11-months	
Participant group 7	Young person	Interviewed at 9-weeks	Interviewed at 7-months	Interviewed at 15-months	*8 interviews*
	Caregiver	Interviewed at 9-weeks	Interviewed at 7-months	Interviewed at 15-months	
	Clinician		Interviewed at 7-months	Interviewed at 15-months	
Participant group 8	Young person	Interviewed at 8-weeks	Interviewed at 4-months	Interviewed at 11-months	*5 interviews*
	*No caregiver identified*				
	Clinician		Interviewed at 4-months	Interviewed at 11-months	
Participant group 9	Young person	Interviewed at 6-weeks	Interviewed at 6-months	Dropped out, discharged at 14-months	*4 interviews*
	*No caregiver identified*				
	Clinician		Interviewed at 6-months	Interviewed at 14-months	
*Total interviews*		*14 interviews*	*21 interviews*	*20 interviews*	*55 interviews*


### Disengagement Processes

**Figure 2:**
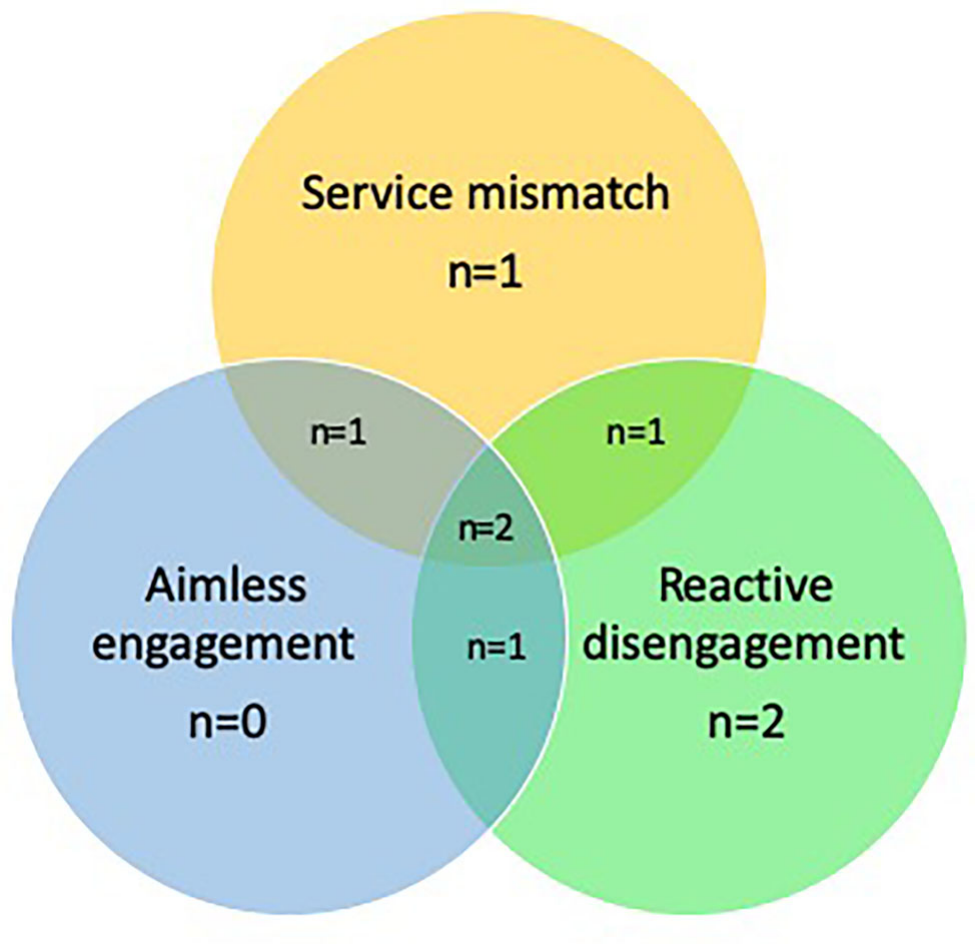
Theme distribution.

Prominent in the data were three processes that led to periods of disengagement from the EIS: a mismatch between service model and individual presentation (service mismatch, affecting five participant groups), a lack of shared purpose (aimless engagement, affecting four participant groups), and responses to individual circumstances (reactive disengagement, affecting six participant groups). Six participant groups experienced more than one type of disengagement process as is demonstrated in **Figure 2**. There were varying reasons for, and successes in service re-engagement.

#### Service Mismatch: A Mismatch Between Service Model and Individual Presentation

Entry into the EIS in each case was precipitated by psychotic symptoms that were assessed as clinically significant and warranted comprehensive early intervention. Young people and caregivers were unsure of what treatment in the EIS would entail and were grateful that clinicians, who they perceived as the experts, could guide them forward in their recovery journey. Clinicians described their early priority as developing a shared understanding of the bio-psycho-social vulnerabilities that may have contributed to the development of the psychotic symptoms.

For two young people (participant group two and participant group eight) in the initial weeks of their contact with the EIS, their psychotic experiences were understood in the context of a communication disorder, a personality disorder, and for both of them, comorbid depressive disorders. The two young people did not easily fit into the FEP EIS model of care as their primary diagnosis was not a psychotic disorder. It is notable that the distress and risks that featured in these cases (e.g., school difficulties, relationship breakdowns, deterioration in mood) were like the distress and risks present across the seven other young people. However, in these two cases, clinician and service attitude shifted from encouraging engagement, to uncertainty about the purpose of engagement and less confidence in knowing how to support the young person:

*“So that's what I struggled with the most, I think. Wanting to engage someone who, his view on reality is fine, and you know, it's not a psychotic picture. It's a more depressive picture, which again, like I said, I don't have a lot of experience in … And engaging him was very challenging, and it still is.”* [Clinician 2, Time-Point 2]

Lack of diagnostic “fit” also became apparent for three more young people over time, as primary diagnoses were determined to be mood or anxiety disorders or both. This led to increasing frustrations for these study participants, including clinicians, as they tried to provide evidence-based practice for FEP which did not align with the young person's primary needs:

*“I suppose the main reflection is, I hope I've summarized, is just around the difficulty of doing client-centered work in a system that doesn't really cater to what might be best for the client. For me, that became a difficult space.”* [Clinician 9, Time-Point 3]

In these cases, intervention shifted from building a therapeutic relationship that was expected to continue for 2 years, to clinicians seeking alternative options, often outside of the EIS, for ongoing support. Clinicians started to disengage from the therapeutic relationship themselves and subsequently, experiences of engagement for all parties became increasingly superficial. This can be contrasted with other case-studies in which young people clearly fitted into the EIS diagnostic model of care (i.e., young person with a diagnosis of schizophrenia), and there were subsequent assertive and complex attempts to maintain engagement with the young person and their caregivers.

Complicating this was a lack of shared understanding across all stakeholders about the reasons for exploring alternative treatment options. For all participant groups where caregivers were involved, there was a strong desire for sustained and active engagement with the EIS to continue regardless of preliminary or actual diagnosis. They described the trauma of navigating systems to find any help for their young person, and there was a sense that the EIS and the clinician were lifeboats they were holding onto firmly. Diagnoses and formulation were helpful in building caregiver and young person understanding of what was happening and helped normalize psychotic and other mental health experiences for them. However, the most significant concern for all was the ongoing disruption to young people's developmental trajectory. This disruption to developmental trajectory was seen across participants, regardless of whether a psychotic disorder was their primary diagnosis. If discharge from the EIS due to lack of diagnostic “fit” was discussed or facilitated at early stages of care, especially when caregivers had not seen an improvement in their young person's presentation, this was accompanied by strong emotions of confusion and uncertainty:

*“Cause I said to [clinician], what can I do with this kid if he's just shut up at home. I mean, he doesn't want anything, but what can you do? He's just there, like, trapped. When I talk … And when I talk to him, he seems to just get a bit hysterical, like, angry, and he doesn't want me to remind him about school. He, he likes it when I talk to him about some things, but not the future.”* [Caregiver 2, Time-Point 3]

Young person reactions to early discharge varied significantly. The young person in participant group two passively engaged with the service throughout his period of care; his engagement was heavily influenced by his caregiver's willingness to commit. As early discharge was discussed and facilitated, he increasingly withdrew from the service. This is in direct contrast to the young person in participant group eight who became increasingly distressed as discharge approached, resulting in discharge plans falling through and her remaining engaged with the EIS.

#### Aimless Engagement: A Lack of Shared Purpose

Clinicians described a second important aspect of the EIS model of care as identifying and working toward mutual goals in the context of a therapeutic relationship. Within the participant groups over the initial 12-months, periods of disengagement notably occurred when goals were misaligned or driven by people other than the young person:

*“He's very blasé about the whole thing—I don't know why I have to come; I don't really get a lot out of coming' … He feels obliged to come because he's in the service and mum makes him come.”* [Clinician 2, Time-Point 2]

Two main factors contributing to this were breakdowns in communication and difficulty accessing supports that the young person required.

When a lack of shared purpose was due to communication breakdowns between different stakeholders, engagement was driven by the dominant voice within the participant group. This dominant voice was predominantly the clinician or the caregiver, with the young people in these cases often describing being unable to talk about how engagement and treatment was for them. An example of this can be found in participant group eight, where the young person felt her needs were not being met but did not want to upset the clinician by articulating this:

“Interviewer: Is there anything else that's not been helpful?

*Young Person: Well, I wouldn't say it's not really helpful but I'm not sure, like, the depression hasn't really been going away. I'm still, like, deciding like when is it gonna go, like, when will it lessen? It's like—It's not, like this is good and stuff, but it's not really doing anything*.

Interviewer: Yeah. How does that feel for you?

*Young Person: Kind of feel like I'm still stuck. And, like, you feel some progress but not very much*.

Interviewer: Yeah. Do you feel able to talk about that with [clinician] or do you feel like you're not able to talk about—?

*Young Person: No, not really. I think it's the one thing that I just want to like, don't want her to feel like she's not enough.”* [Young Person 8, Time-Point 2]

This meant that the purpose of engagement was driven by the clinician and focused predominantly on addressing social connectedness rather than targeting symptoms of depression. The decreasing participation of the young person due to this communication breakdown meant that care, over time, became increasingly paternalistic. While the young person continued engaging with the EIS, it was only when treatment for depression commenced that the engagement was identified to be more useful by the young person and the power imbalance lessened.

Another example of this type of communication breakdown was found in in participant group five, as articulated by the clinician:

*“And he'd disengage occasionally here, like he'd disappear for a few weeks or be hard to contact. And I think that was, that's his kind of pattern, if maybe things were getting too much or he didn't want to … I don't think [young person] would ever acknowledge that or may not have been aware of that, because he's a people pleaser, you know, yeah, I think, you know, doesn't want to—he always used to say - I'd let you know, I'd tell you if things weren't going how I'd want them to be going in sessions, I'm finding them really helpful. But my sense is that probably [he] wouldn't actually have if that makes sense.”* [Clinician 5, Time-Point 3]

As this type of communication breakdown occurred, engagement appeared to fade away and this only shifted if there was a clear reason for re-engagement, such as relapse of symptoms or psychosocial crisis.

Difficulties in accessing supports or unclear expectations on what the EIS could provide also led to periods of aimless engagement, as demonstrated by participant group one. The young person and their caregiver identified an urgent need for support with accommodation and finances. Lack of stable accommodation and fluctuating income for illness-related reasons were impeding other aspects of recovery, such as re-engaging in vocational activities. However, the capacity to influence these specific goals sat outside the clinician's influence, leading to a rupture in the therapeutic relationship and a lack of clear purpose for ongoing engagement:

*“I think [young person] got a bit frustrated. You know he said he wanted a different accommodation and I think maybe had a false expectation that I could just find him a new house and when that wasn't happening, I wonder if he maybe didn't see as much value in some of the sessions. I'm just kinda speculating but I think that might have contributed.”* [Clinician 1, Time-Point 2].

As his clinician was attempting to support him with his psychosocial needs by discussing them in sessions and providing advice, the young person had difficulty distinguishing between what could be provided from within the EIS and what required linkages and support from external services. For this young person over the 12-months, there were repeated clear periods of disengagement from the service as his more practical needs were not able to be met, followed by re-engagement due to relapse of acute symptoms.

#### Reactive Disengagement: Responses to Individual Circumstances

A more intense type of disengagement process was experienced when there was a clear change in circumstance. For some participants, this was due to positive reasons, such as returning to work or school. Engagement with the EIS became a second priority that young people would follow through with if it did not impact on their primary priority. Unfortunately, the constraints of the EIS (i.e., only operating during business hours) meant that disengagement often occurred in these circumstances:

*“Because I'm starting work and I still want to see a psychologist. I reckon one thing I could change about [EIS] is, not changing but adding, like you guys are open like 2, 3 hours on a Saturday or a Sunday. So, for people that might be busy during the week, they can still have a session. And it won't be that long because there won't be that many people that can't do it during the week.”* [Young Person 3, Time-Point 3].

Disengagement in this circumstance was not only led by the young person, but could also be clinician driven, with some young people being discharged from the EIS to a service with more flexible opening hours:

*“So, over the last 6 months, YP got a full-time job, which is fantastic. And it became quite difficult for her to attend appointments around the hours that she was working. So, she disengaged for a very long time, probably the majority of the last 6 months or so. Initially there was that contact maintained with mum and attempts to get her in for reviews, but it was clearly becoming something that wasn't really working for either party. So, we had a conversation and decided to proceed discharge planning.”* [Clinician 9, Time-Point 3].

Another cause of reactive engagement was when there was a service-initiated break in the therapeutic relationship. Of the nine case-studies, six young people and most caregivers specifically discussed the importance of consistency. The therapeutic relationship between all parties deepened over time and was unanimously described to be the main positive influencer of engagement. Any change in key clinician had marked, negative impacts on service engagement. At the 12-month time-point, two young people had experienced changes in key clinicians and three young people had further changes pending. Reasons for this included clinician resignations and junior clinicians stepping into key clinician roles for time-limited periods, as part of graduate training programs.

The impact of change in key clinicians was associated with a sense of loss. One young person (participant group one) had an itinerant lifestyle, resulting in him frequently moving between catchment areas. This meant that the EIS clinic he received care from changed at the 6–9-month period and subsequently his key clinician changed. From his perspective, he had built a trusting relationship with his first clinician and he felt immense loss at the end of that relationship. This is despite the fact that from the initial clinician's perspective, therapeutic engagement was limited due to his infrequent attendance and crisis driven contacts:

*“Like, [Clinician 1a], I probably talked more to and let more out, but I can't with [Clinician 1b]. Just cause, I got sort of trust in [first clinician] … Now I can't trust anyone, not even family.”* [Young Person 1, Time-Point 3].

Trust was built with the second clinician over time with consistency in approach and eventually it was identified as stronger than with the first clinician. Despite this, the young person continued to move frequently and at 12-months, was in the process of being discharged to another service, more local to him.

A permanent change in clinician also occurred for participant group four, in which the young man had most of his appointments at his house due to poor capacity to attend clinic appointments. His change in clinician occurred at the 9–12-month period. The transition was difficult, with the new clinician struggling to establish contact due to the young person's chaotic living and social circumstances. On reflection on the change in clinicians, the young person and their caregiver missed the connection with the first clinician, but saw the service as a team:

*“We think they're lovely too. I can't say—they're all the same, you know what I mean? Or like, everyone's really nice and they put in a real lot of work for [young person]. Do you know what I mean? So, it's sort of no different what worker he's had, they've all been lovely. You know, and they care about him. Yeah, so it's good.”* [Caregiver 4, Time-Point 3].

This family unit had multiple health and social services involved throughout their lives, which had appeared to normalize the experience of key workers changing on a frequent basis. This meant that change in key clinician did not necessarily initiate disengagement, but it did lead to periods of reduced quality of engagement and a loss of momentum in recovery.

## Discussion

We aimed to understand what initiates periods of disengagement in the first year of connection with an EIS. Three major processes were identified that led to periods of service disengagement: a mismatch between service model and individual presentation (service mismatch), a lack of shared purpose over time (aimless engagement), and responses to individual circumstances (reactive disengagement). Understanding and seeking to address these disengagement processes is critical, as engagement with an EIS following a FEP is often the first contact individuals have with specialist mental health services. Experiences of engagement therefore not only impact initial recovery from the FEP but also establish expectations for longer-term engagement and influence openness for future help seeking (12, 17).

In this study, which attempted to follow a real-life cohort of young people through their first 12-months with an EIS, we found that eight of the nine participants experienced some form of disengagement from the EIS, which is higher than the 6–60 percent identified in previous literature (8). However, for most participants, these experiences of disengagement were not absolute and occurred with varying levels of intensity. For both service mismatch and aimless engagement, engagement deteriorated over time if the precipitator/s did not change. With reactive disengagement, experiences occurred in the context of a specific precipitator and were observed to happen faster and more intensely. There was an important temporal element to all the disengagement processes that highlights the need for clinicians and services to be attune to the engagement experience of young people and their caregivers throughout the entire episode of care, and not only in the beginning. There may be an assumption in the FEP EIS model of care of a pre-determined pathway (i.e., assessment, psychoeducation, clinical formulation, treatment, and discharge) (18). However, the model must maintain capacity for increased intensity and flexibility as risk periods for disengagement occur. One important example is the need to more actively and intensively re-engage a young person if their key clinician changes. It is imperative that care following a FEP is individualized, responsive, and flexible over time.

In this study, all young people experienced as significant the impacts of psychotic symptoms on personal, vocational, and relational aspects of their lives. Young people and caregivers were focused on psychosocial level of distress and disruption, not on diagnostic differences. However, the EIS appeared to prioritize their focus and resources on those young people who would be diagnostically classed as having a major mental illness (i.e., schizophrenia, schizoaffective disorder, bipolar affective disorder). This is most likely a consequence of the broader service, policy, and societal influences on engagement (14, 19).

Contemporaneous understanding of psychotic symptoms is that they present on a continuum across a variety of mental health disorders and are a predictor of greater illness severity (20). The ability to accurately predict the outcome of a FEP is extremely limited (21). Most people with psychotic symptoms will never transition to, or meet the diagnostic criteria of, schizophrenia. However, the translation of this understanding to clinical practice remains limited. This may be a reflection of EIS models of care being driven by a focus on first-episode schizophrenia, rather than truly providing for the broad reality that is FEP. Psychotic symptoms are often judged for their level of importance within the context of other presenting concerns. For example, when a personality disorder is concurrently diagnosed, psychotic symptoms are often perceived by health professionals to not be as important to treat as those experienced by people with, for example, schizophrenia (22). The lack of attention to people whose psychotic symptoms do not, over time, align with a schizophrenia diagnosis is a significant shortcoming of EISs.

Actively committing to translating the early intervention approach to a broader spectrum of mental health distress is an important action needed for youth mental health reform (23). There are substantial benefits to the early intervention approach when compared with treatment as usual (5). However, there are clear areas for improvement in meeting the needs of young people attending EISs for FEP, and alternative models should be explored. Transdiagnostic approaches, such as clinical staging models, may reduce the potential for disengagement processes that are shown to occur due to a mismatch between the person and diagnostically driven approaches (24–27). Clinical staging models comprise of stages ranging from stage 0 (asymptomatic individuals at risk of mental illness) through to stage 4 (severe, persistent, and unremitting illness). Clinical stage is based on degree of severity, persistence, distress and functional impairment, and treatment is personalized accordingly.

Providing care within one service as young people transition through the stages of illness and distress would be transformational for EISs, as it would promote continuity of care and ensure that the benefits found within early intervention are accessible to all young people who need them. This would also be in line with the Australian Productivity Commission (1) which recommends a stepped care approach to mental health treatment; that is, access to health care in line with the person's individual treatment and care needs. A true stepped care model provides support on a scale from self-management, to low-high intensity care, to complex care. Given the psychosocial distress young people and caregivers experienced at entry to the EIS, this would allow needs-based treatment to be provided, regardless of the person's diagnosis. Discharge could then occur flexibly at a mutually agreed upon timeframe, rather than a pre-determined 2 years. Consideration could also be given to periods of less-frequent and/or young-person initiated contact prior to a formal discharge, which would afford young people increased flexibility and ownership in their final disengagement process.

Moving to a more inclusive, transdiagnostic model of care would also address another current and pressing issue in mental health, the “missing middle” (1). The concept of the missing middle describes the group of people who are too unwell for primary care services but do not meet the diagnostic or risk criteria for entry to tertiary level mental health services. Mental health services specifically for young people help lessen this division, as compared to traditional mental health services, but gaps in service care remain apparent (23). If young people are not offered individualized support that is responsive and flexible to their needs, then symptoms and risk may deteriorate while associated distress and impacts on psychosocial functioning become more apparent. In this study, those young people whose psychotic symptoms were associated with depression, anxiety, personality or neurodevelopmental constructs appeared to experience an impoverished form of engagement with the service. There is the risk that those young people who did not fit the service model, and were to be discharged from the EIS early, may become part of the missing middle.

Despite variances in preliminary or actual diagnoses, most young people overall were eager to work with their clinician on addressing social and psychological areas of recovery. Disengagement occurred when goals were not clearly articulated, when clinicians were questioning the usefulness of interventions, when goals could not be supported by the EIS or when goals were service led. Mutual goal setting, with clearly outlined roles and responsibilities, therefore remains an important aspect of services delivery in EISs. Open communication about this is critical. The use of models such as shared and supported decision making to discuss treatment options, and therefore facilitate conversations clearly articulating the purpose for engagement, and the roles and responsibilities within this, cannot be understated (28).

Attention also needs to be given to the mental health workforce and resources. This includes the urgent and ongoing need for support and supervision of clinicians who may be struggling to engage with a young person or their caregivers. Continuity of care remains difficult to achieve while there are known shortages in the workforce. Training requirements often mean that there is a schedule of students or junior clinicians who step into more senior and demanding clinician roles for defined periods of time. Services that offer time-limited periods of care also necessitate the need for transitions and these result in disruptions to care (23). This highlights the importance of giving priority to continuity of relationship in service planning and the need for investing more clinician time at any transition points. However, there are also complexities in balancing the need for continuity of care and matching clinician skills with young person needs. Each allied health discipline specializes in different approaches, and within this, each individual clinician may choose to train in other nuanced approaches (29). Teams must be enabled to reflect on this early in engagement to minimize disruptions and maximize potential benefits, and to ensure that all people receive the core treatment components of the EIS model of care.

Poor attention to practical aspects of mental health services, such as accessibility and opening hours continue to pose a barrier for many young people accessing them. The impact of this is significant, causing at least a disruption in care, but more often partial or complete service disengagement. Online treatment, telehealth, or blended approaches (face-face, telehealth and online support) should be actively considered as alternative treatment options to facilitate access to treatment regardless of time of day, especially while services continue to operate during limited hours (1, 23). This would also allow young people to engage with the EIS according to their personal preferences and needs.

This study highlights the importance of further commitment and research into re-evaluating the mental health service system as a whole. Research into transdiagnostic models that facilitate care for complex and evolving mental health disorders will help inform future service directions. There is also a critical need for translational research that evaluates experiences of such models of care from the perspectives of all involved, taking into account barriers or challenges to both implementation and continuing care provision.

### Strengths and Limitations of the Study

This is the first longitudinal study on engagement with EISs that represents the perspectives of young people, their caregivers and their clinicians. It is therefore uniquely placed to highlight service challenges and experiences of disengagement incorporating the perspectives of all stakeholders. There was diversity in our cohort, with participants coming from a range of ethnic and social backgrounds and presenting with a range of psychosis-related diagnoses. Interestingly, there were no clinicians from a nursing background associated with the young people in the first 12-months of this study, despite nurses commonly working within EISs. This limits the comparability of the nursing experiences of engaging with young people in EISs. There were also no male caregivers identified and male caregivers were notably absent from participants' stories. Engagement (or disengagement) of male caregivers with EISs is an area that would benefit from further exploration and research. Further limitations include that the researchers are all of white, middle-class backgrounds and work within the healthcare system either as researchers or clinician-researchers. They therefore hold value in the concept of early intervention for mental health conditions. The lead researcher was associated with the EIS for the initial period of data collection and despite careful consideration to dual relationships and confidentiality, this may have impacted on both the participants' inclination to openly reflect and the lead researcher's approach to collecting, analyzing, and interpreting the data. It should also be noted that although the principles of EISs internationally are very similar, there are large variabilities in the way services are delivered. The findings of this study may therefore be site-specific.

### Conclusions

Our findings challenge assumptions about diagnostically driven models of care and have important implications for clinicians and policy makers. Disengagement from services is experienced by individuals but may often be a consequence of inadequate mental health service systems. Given the complex and evolving nature of psychotic symptoms, and the uncertainty surrounding eventual diagnosis and outcome, early intervention models of care should focus on individual needs. This will require mental health services to shift away from diagnosis, instead understanding mental health distress as presenting on a continuum. This will facilitate services for people who present with transdiagnostic symptoms and syndromes and will reduce barriers to the “missing middle” accessing care. Societal and political attention should be directed toward adequately resourcing services to be able to provide this, while also taking into account the importance of continuity of care, accessibility, and communication. 

## Data Availability Statement

The datasets presented in this article are not readily available because of the sensitive and personal information disclosed during the qualitative interviews. De-identified data are available from the corresponding author on reasonable request. Requests to access the datasets should be directed to rachel.tindall@orygen.org.au.

## Ethics Statement

This study involved human participants. As such, it was reviewed and approved by Melbourne Health Human Research Ethics Committee. Written informed consent to participate in this study was provided by all participants. For participants under the age of 18, written informed consent was provided by the young person and the participants' legal guardian/next of kin. Written informed consent was obtained from individual(s) and, if applicable, minor(s)' guardian/next of kin, for the publication of any potentially identifiable images or data included in this article.

## Author Contributions

RT collected and analyzed the participant data and was a major contributor in writing the manuscript. MS, KA, and BH analyzed participant data and were major contributors in writing the manuscript. All authors contributed to the article and approved the submitted version.

## Funding

This project was supported by The Australian Government Research Training Program Scholarship, HOSPIRA, The Windermere Foundation Doctoral Scholarship in Health (Nursing) Program and The National Health and Medical Research Council of Australia.

## Conflict of Interest

The authors declare that the research was conducted in the absence of any commercial or financial relationships that could be construed as a potential conflict of interest.
